# Disadvantage of survival outcomes in widowed patients with colorectal neuroendocrine neoplasm: an analysis of surveillance, epidemiology and end results database

**DOI:** 10.18632/oncotarget.13078

**Published:** 2016-11-04

**Authors:** Jing Li, Ying Wang, Fang Han, Zhu Wang, Lichun Xu, Jiandong Tong

**Affiliations:** ^1^ Department of Oncology, Yangzhou NO.1 People's Hospital, The Second Clinical School of Yangzhou University, Yangzhou, Jiangsu Province, China; ^2^ Research Center of Cancer Prevention and Treatment, Medical College of Yangzhou University, Yangzhou, Jiangsu Province, China

**Keywords:** colorectal neuroendocrine neoplasms, marital status, prognostic analysis

## Abstract

Marital status correlates with health. Our goal was to examine the impact of marital status on the survival outcomes of patients with colorectal neuroendocrine neoplasms (NENs). The Surveillance, Epidemiology and End Results program was used to identify 1,289 eligible patients diagnosed between 2004 and 2010 with colorectal NENs. Statistical analyses were performed using Chi-square, Kaplan–Meier, and Cox regression proportional hazards methods. Patients in the widowed group had the highest proportion of larger tumor (>2cm), and higher ratio of poor grade (Grade III and IV) and more tumors at advanced stage (P<0.05). The 5-year cause specific survival (CSS) was 76% in the married group, 51% in the widowed group, 73% in the single group, and 72% in the divorced/separated group, which manifest statistically significant difference in the univariate log-rank test and Cox regression model (P<0.05). Furthermore, marital status was an independent prognostic factor only in Distant stage (P<0.001). In conclusion, patients in widowed group were at greater risk of cancer specific mortality from colorectal NENs and social support may lead to improved outcomes for patients with NENs.

## BACKGROUND

Neuroendocrine neoplasms (NENs) originated from neuroendocrine cells present throughout the body and are capable of producing a variety of biogenic amines. NENs of the large intestine account for 20% of all NENs and are most commonly found in the rectum [1]. The data from Surveillance, Epidemiology, and End Results (SEER) database indicated that the age-adjusted incidence of NENSs rose from 1.9 to 5.25 cases per 100,000 people between 1973 and 2004 [2]. Diagnosed incidence of NENs is predicted to continue rising at a faster rate than other malignant neoplasms [3]. Although with advances in surgical therapy and the introduction of new treatment strategies, survival outcomes of NENs remain unchanged in the past 30-year [4]. This may be due to late diagnosis and lack of effective prognostic biomarkers.

In a variety of malignancies, the effect of marital status is being increasingly recognized as a determinant of stage at diagnosis, as well as a determinant of the prognosis of treated cancer [5–11]. A study based on SEER database demonstrated that patients of unmarried are always accompanied with higher risk of advanced tumor stage, undertreatment, and cancer related death in top ten causes of cancer-related death [5]. However, to our knowledge, the correlation between marital status and survival of patients with NENs has not been previously studied. In the present study, we used data from the SEER database of patients diagnosed between 2004 and 2010 to investigate what aspects of marital status affect survival of patients with colorectal NENs in detail.

## RESULTS

### The characteristic of patients in SEER database

We identified 1,289 eligible patients during the 7-year study period, including 617 male and 672 female patients. Of these, 808 (62.7%) were married, 119 (9.2%) were widowed, and 226 (17.5%) had never been married. The 9 (0.7%) patients in separated subgroup and 127 (9.9%) in divorced subgroup were incorporated into the divorced/separated group in the present study. Patients in the widowed group had the highest proportion of female, more common of colon as primary site, more prevalence of elderly patients (>50 years), higher percentage of larger tumor size (>2cm), higher ratio of poor grade (Grade III and IV) and more tumors at advanced stage (Distant and Regional) (P<0.001). The percentage of surgery performed was comparable between the married and widowed groups (P=0.367). The characteristics of patients are summarized in Table 1.

**Table 1:** Baseline demographic and tumor characteristics of patients in SEER database

Characteristic		Married	Windowed	Single	Divorced/separated	Total	Chi-square(χ^2^)	P-value
		n=808 N (%)	n=119 N (%)	n=226 N (%)	n=136 N (%)	n=1289 N (%)		
Sex							55.037	<0.001
	Male	438(54.20)	26(21.8)	106(46.90)	47(34.60)	617(47.90)		
	Female	370(45.80)	93(78.20)	120(53.10)	89(65.40)	672(52.10)		
Age							89.456	<0.001
	<=50	216(26.70)	2(1.70)	110(48.70)	35(25.70)	363(28.20)		
	>50	592(73.30)	117(98.30)	116(51.30)	101(74.30)	926(71.80)		
Race							44.221	<0.001
	White	606(76.70)	97(81.50)	160(71.40)	95(70.40)	958(75.60)		
	Black	87(11.00)	14(11.80)	53(23.70)	31(23.00)	185(14.60)		
	Other	97(12.3)	8(6.70)	11(4.90)	9(6.70)	125(9.90)		
Primary site							24.592	<0.001
	Colon	437(54.10)	89(74.80)	117(51.80)	89(65.40)	732(56.80)		
	Rectum	371(45.90)	30(25.20)	109(48.20)	47(34.60)	557(43.20)		
Grade							55.811	<0.001
	Grade I	494(61.10)	44(37.00)	137(60.60)	77(56.60)	752(58.30)		
	Grade II	121(15.00)	13(10.90)	36(15.90)	14(10.30)	184(14.30)		
	Grade III	160(19.80)	51(42.90)	36(15.90)	31(22.80)	278(21.60)		
	Grade IV	33(4.10%)	11(9.20)	17(7.50)	14(10.30)	75(5.80)		
Tumor size							26.463	<0.001
	<=2cm	438(54.20)	38(31.90)	126(55.80)	58(42.60)	660(51.20)		
	>2cm	370(45.80)	81(68.10)	100(44.20)	78(57.40)	629(48.80)		
Histological stage							27.481	<0.001
	Localized	406(50.20)	37(31.10)	129(57.10)	56(41.20)	628(48.70)		
	Distant	185(22.90)	43(36.10)	52(23.00)	38(27.90)	318(24.70)		
	Regional	217(26.90)	39(32.80)	45(19.90)	42(30.90)	343(26.60)		
Surgery							3.165	0.367
	Not performed	67(8.30)	11(9.20)	26(11.50)	9(6.60)	113(8.80)		
	Performed	741(91.70)	108(90.80)	200(88.50)	127(93.40)	1176(91.20)		
CSS status							33.460	<0.001
	Live	640(79.21)	66(55.46)	178(78.76)	105(77.21)	989(76.73)		
	Dead	168(20.79)	53(44.53)	48(21.24)	31(22.79)	300(23.23)		

### Impact of marital status on CSS in NENs

**Figure 1:**
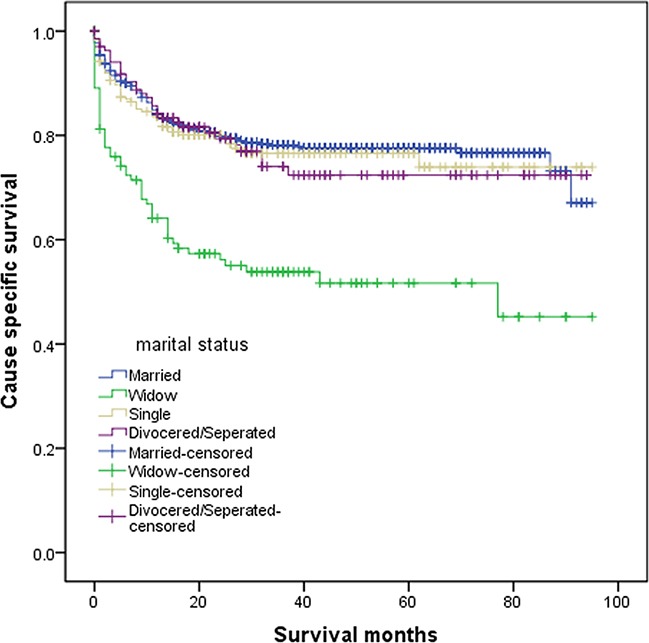
Survival curves of patients with colorectal neuroendocrine tumor according to marital status The overall 5-year CSS was 76% in the married group, 51% in the widowed group, 73% in the single group, and 72% in the divorced/separated group, the difference of which was significantly different according to the univariate log-rank test (P<0.001).

Of all, 300 patients died of NENs and 69 (5.35%) patients who died of other reasons were censored for analysis. The overall 5-year CSS was 76% in the married group, 73% in the single group, 72% in the divorced/separated group, and 51% in the widowed group (P<0.05). Patients in the widowed group always had the lowest survival rate when compared with patients in other groups. Patients in widowed group had a 24% reduction in 5-year CSS compared with married patients (P<0.001), and the difference between the never married and divorced/separated group was not of significance (Table 2) (Figure 1). Additionally, in univariate analysis, elderly patients (P<0.001), primary site at colon (P<0.001), White race (P=0.004), poor or undifferentiated tumor grade(Grade III/IV) (P< 0.001), tumor size above 2cm (P<0.001), having distant metastasis or lymph node metastasis(P<0.001) and no surgery performed (P<0.001) were negatively correlated with survival outcome, while there is no significant difference in sex (P=0.328)(Table 2). Factors with significance in univariate analysis were incorporated into multivariate Cox regression analysis and six variables were verified as independent predictors, including Grade [grade II, hazard ratio (HR) 3.026, 95% confidence interval (CI) 1.786-5.127; grade III, HR 9.761, 95% CI 6.5-14.658; grade IV, HR 13.062, 95% CI 8.204-20.797), tumor size (tumor size above 2cm, HR 1.84, 95% CI 1.17-2.892), histological stage (distant, HR 12.954, 95% CI 7.07-23.737; regional, HR 3.496, 95% CI 1.88-6.5), primary site (rectum, HR 0.661, 95% CI 0.469-0.931), surgery (surgery performed, HR 0.637, 95% CI 0.452-0.896), and marital status (widowed, HR 1.734, 95%CI 1.259-2.39; single HR 1.365, 95% CI 0.972-1.917; divorced/separated, HR 0.694, 95% CI 0.467-1.032) (Table 3).

**Table 2:** Univariate analysis of cause specific survival in patients with colorectal neuroendocrine neoplasms

Variable	N	5-year CCS (%)	Log rank test (χ2)	P-value
Sex			0.955	0.328
Male	617	72		
Female	672	74		
Age			23.294	<0.001
<=50	363	82		
>50	926	70		
Primary site			120.706	<0.001
Colon	732	60		
Rectum	557	90		
Race			10.918	0.004
White	958	70		
Black	185	82		
Other	125	80		
Marital status			43.411	<0.001
Married	808	76		
Windowed	119	51		
Single	226	73		
Divorced/separated	136	72		
Grade			768.64	<0.001
Grade I	752	94		
Grade II	184	82		
Grade III	278	28		
Grade IV	75	14		
Tumor size			297.924	<0.001
<=2cm	660	95		<0.001
>2cm	629	51		
Histological stage			652.227	<0.001
Localized	628	98		
Distant	318	23		
Regional	343	75		
Surgery			37.949	<0.001
Not performed	113	52		
Performed	1176	75		

**Table 3:** Multivariate analysis of cause specific survival in patients with colorectal neuroendocrine neoplasms

Variables	Hazard Ratio	95%CI	P-value
Race			0.786
White	1	reference	
Black	1.085	0.732-1.609	0.684
Other	1.144	0.733-1.787	0.553
Grade			<0.001
Grade I	1	reference	
Grade II	3.026	1.786-5.127	<0.001
Grade III	9.761	6.5-14.658	<0.001
Grade IV	13.062	8.204-20.797	<0.001
Marital status			<0.001
Married	1	reference	
Widowed	1.734	1.259-2.39	0.001
Single	1.365	0.972-1.917	0.072
Divorced/separated	0.694	0.467-1.032	0.071
Age			0.596
<=50	1	reference	
>50	1.09	0.792-1.502	0.596
Tumor size			0.008
<=2cm	1	reference	
>2cm	1.84	1.17-2.892	0.008
Histological stage			<0.001
Localized	1	reference	<0.001
Distant	12.954	7.07-23.737	<0.001
Regional	3.496	1.88-6.5	<0.001
Primary site			0.018
Colon	1	reference	
Rectum	0.661	0.469-0.931	0.018
Surgery			0.01
Not performed	1	reference	
Performed	0.637	0.452-0.896	0.01

**Figure 2:**
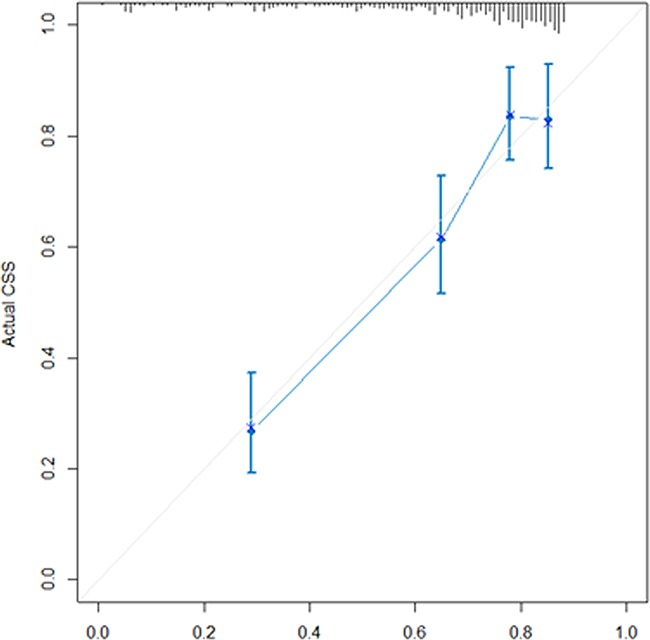
Calibration plot for Table 3 The reference line is 45 degree and indicates perfect calibration.

The C-index of the Cox regression model is 0.869 (95%CI: 0.851-0.886) and the calibration plot indicated perfect calibration (Figure 2).

## DISCUSSION

The conclusion in the present study demonstrates the first available research of the impact of marital status on survival of patients with colorectal NENs. The impact of marital status is independent of age, grade, histological stage, tumor size, primary site and surgical treatment. These results suggest that patients in unmarried group are always accompanied with metastatic cancer and high incidence of death from colorectal NENs. Besides, widowed patients always had worse survival than other patients, which was verified as an independent predictor in NENs.

An important aspect of the present study is making detailed analysis of unmarried patients and identifying widowed patients as a subgroup who have the poorest survival outcomes. Although the impact of marriage has been studied in many cancers, fewer of them pay attention to the heterogeneity of unmarried patients. In the present study, we found unmarried patients with NENs was a heterogeneous group. Single and divorced/separated patients have similar 5-year CSS with married patients, and only widowed patients had significantly worse survival outcome than patients in other groups.

Two hypotheses have always been used to explain the relationship between health and marital status. First, unmarried patients are often diagnosed with more advanced tumor stage, and they are also more likely to receive insufficient treatment. In the present study, widowed patients have highest proportion of distant histological stage while with lowest percentage of localized disease. In addition, widowed patients are more prevalence of poor tumor differentiation (Grade III/IV). Conversely, widowed patients are more common of White in race. Previous studies showed that patients in White race always have good insurance status and relatively better survival outcomes than others [12–14], which suggests that marital status may play a critical role in survival outcomes of patients with colorectal NENs.

The other hypothesis for the impact of marital status on CSS considered marriage as a means of social support. Cancer diagnosis and treatment induce acute and chronic stress, which may cause poor adherence to medical interventions [15]. Meta-analyses confirmed that patients with depression have an increased death rates with range from 19% to 39% [16, 17]. A behavioral research suggests that cognitive, behavioral and social factors are of great importance in promoting the customization to active treatment and throughout cancer survivorship, which forms the fundamentals for the application of many psychological and social supportive interventions in patients with cancer [18]. The loss of social support or the inability to cope with stress in the widowed group seems very apparent, which may lead to excess mortality [11].

There are several limitations of our present study. First, we did not consider changes of marital status that may occurred during follow-up, which may influence long time survival rate. Thus, our findings may underestimate the protective effect of marriage on colorectal NENs outcomes [19]. Second, SEER database lacks information of education, income status, life style, insurance status, socioeconomic status and quality of marriage, which might have impacts on the survival outcomes and confound the multivariate Cox regression analysis. For example, marital distress has long term immune consequences and enhances the risk of a variety of health problems [20]. Third, SEER database lacks the information of adjuvant therapy. Fourth, it is likely that some individuals cohabitated without marriage and were categorized as unmarried in SEER database. Actually, patients with such partnership are likely to have better survival outcomes than the unmarried patients, which may bias our results [5].

In conclusion, our study found that widowed patients are associated with higher risk for diagnosis with a later stage of colorectal NENs and for worse cancer related survival outcomes than others. This finding indicated social support may greatly amend traditional therapy and may improve outcomes in widowed patients.

## MATERIALS AND METHODS

### Patient selection in the SEER database

The SEER database is a population-based cancer registry across several disparate geographic regions. The SEER research data includes cancer incidence as well as age, sex, race/ethnicity, year of diagnosis, marital status, Tumor-Node-Metastasis (TNM) stage, and tumor grade. It contains no identifiers and is widely used for studies of the relationship between marital status and survival outcomes of patients with cancer [5, 11, 21]. The exact dataset we used for this analysis was SEER Program (www.seer.cancer.gov) Research Data (2004–2010)

The National Cancer Institute's SEER*Stat software (Surveillance Research Program, National Cancer Institute SEER*Stat software, www.seer.cancer.gov/seerstat)(Version 8.1.2) was used to identify patients whose pathological diagnosis was colorectal NENs between 2004 and 2010. We identified colorectal neoplasms cases using ICD-O-3 histology codes for NENs (8240, 8241, 8246, 8249) and selected patients with primary site labeled as C18.0-C19.9 (colon) and C20.9 (rectum). Race was categorized into White, Black, and other (American Indian, AK Native, Asian and et al) as provided by the SEER data. Patients were excluded if age at diagnosis was less than 18 years, or if they had undefined marital status or tumor grade, of if they had missed therapy information (surgery performed or not), unknown cause of death or unknown survival months.

### Ethics statement

This study was based on the free public SEER database (www.seer.cancer.gov/seerstat). We have got permission to access the research data file in SEER program and the reference number was 11756-Nov2013. The study was approved by the Review Board of The Second Medical School of Yangzhou University, Yangzhou, China.

### Statistical analysis

Detailed information regarding patient (age, sex, race, marital status) -, tumor- related variables (tumor size, histological stage, primary site, and tumor grade), therapy information (surgery performed or not), and survival information [SEER cause cancer specific survival (CSS)] was retrieved from SEER database. The patients were divided into two age groups: ≤50 years (young) and >50 years (old). Marital status is coded as married, divorced, widowed, separated, and never married at the time of diagnosis. Individuals in the separated and divorced group were clustered together as the divorced/separated group in the present study. According to the SEER staging system, tumors that remain in situ or confined to the organ are regarded as localized, while those that locally invade or metastasize to regional lymph nodes are considered to be regional, and those that travel to distant organs are categorized as distant. Furthermore, there is no accepted uniform grading system for malignant NENs to now. Pathologists in the United States before 2010 typically used the term“carcinoid tumor” to denote well-differentiated NENs (GI) and the term “atypical carcinoid”to describe a moderately differentiated carcinoid (GII). Poorly differentiated tumors are generally classified as GIII tumors, and undifferentiated anaplastic tumors are classified as GIV tumors [22].

Chi-square tests were used to examine the association between marital status and other variables. The Kaplan-Meier method was used to estimate survival curves. Differences between the curves were analyzed by log-rank test. Multivariable Cox regression models were built for analysis of risk factors for survival outcomes. Exact 95% CIs for proportions were calculated. The primary endpoint of this study was CSS, which was calculated from the date of diagnosis to the date of cancer specific death. Deaths attributed to colorectal NENs were treated as events and deaths from other causes were treated as censored observations. The 5-year CSS rate was estimated from Kaplan-Meier curves. All of the statistical analyses were done using the statistical software package SPSS for Windows, version 17 (Chicago: SPSS Inc, USA). Statistical significance was set at two-sided (P < 0.05).
